# Exploring sarcasm detection in amyotrophic lateral sclerosis using ecologically valid measures

**DOI:** 10.3389/fnhum.2013.00178

**Published:** 2013-05-21

**Authors:** Mathew Staios, Fiona Fisher, Annukka K. Lindell, Ben Ong, Jim Howe, Katrina Reardon

**Affiliations:** ^1^School of Psychological Science, La Trobe UniversityMelbourne, VIC, Australia; ^2^Calvary Health Care BethlehemMelbourne, VIC, Australia

**Keywords:** amyotrophic lateral sclerosis (ALS), frontotemporal dementia (FTD), social cognition, emotion recognition, executive dysfunction

## Abstract

Amyotrophic lateral sclerosis (ALS) is a rapidly progressive condition involving degeneration of both upper and lower motor neurons. Recent research suggests that a proportion of persons with ALS show a profile similar to that of frontotemporal dementia (FTD), with this group of ALS patients exhibiting social cognitive deficits. Although social cognitive deficits have been partially explored in ALS, research has yet to investigate such changes using ecologically valid measures. Therefore, this study aimed to further characterize the scope of social cognitive and emotion recognition deficits in non-demented ALS patients using an ecologically valid measure of social cognition. A sample of 35 ALS patients and 30 age-and-education matched controls were assessed using the Addenbrooke's Cognitive Examination, the Brixton Spatial Anticipation Test, and The Awareness of Social Inference Test, where participants were required to discriminate between various emotions and decipher socially challenging scenarios enacted in video vignettes. Participants with ALS showed significant difficulties in recognizing both sarcastic and paradoxical sarcastic statements, but not sincere statements, when compared to controls. After controlling for executive difficulties, ALS patients still displayed significant difficulties on tasks that assessed their comprehension of both sarcastic and paradoxical sarcastic statements. The inability to read social cues and make social inferences has the potential to place significant strain on familial/interpersonal relationships in ALS. The findings of this study highlight the importance of employing a broader range of neuropsychological assessment tools to aid in early detection of frontal lobe impairment in non-demented ALS patients.

Amyotrophic lateral sclerosis (ALS), the most common variant of motor neuron disease, is a rapidly progressive condition involving degeneration of both upper and lower motor neurons (Mitsumotot et al., 2005). The traditional assumption that ALS spares cognitive function is now considered to be incorrect (Strong et al., 2009; Phukan et al., 2012). Research consistently reports functional and structural changes in the frontotemporal region, manifesting as executive dysfunction, including behavioral dysinhibition; a pattern of deficits similar to that observed in frontotemporal dementia (FTD; Lomen-Hoerth et al., 2000; Ringholz et al., 2005; Murphy et al., 2007; Meier et al., 2010; Girardi et al., 2011).

Current formulations of executive functions recognize them to be a distinct cognitive system that regulates other cognitive processes. They include such cognitive constructs as attention, working memory, multi-tasking, mental flexibility, initiation/responsiveness, problem solving, verbal reasoning, inhibition, and self-monitoring of one's own behavior (Alvarez and Emory, 2006). Studies (Abrahams et al., 2000; Gibbons et al., 2007) examining executive dysfunction in ALS have employed a wide range of assessment tools (i.e., verbal fluency, Wisconsin Card Sorting Test). However, the administering of cognitive measures is often problematic in ALS due to upper limb dysfunction and speech impairment (i.e., dysarthria and dysphagia), with studies having to make adjustments to test administration, scoring, and increasing the duration of time taken to complete such tasks (Abrahams et al., 2000). In contrast a relatively new measure of executive functioning, the Brixton Spatial Anticipation Test (Burgess and Shallice, 1997), measures a person's ability to detect a rule, to follow it, and to switch to a new rule. This executive task does not require a verbal or complex motor response by the patient, and thus circumvents administrative issues faced by researchers attempting to measure executive decline in ALS.

More recently, ALS research (Meier et al., 2010; Girardi et al., 2011) has described a similar profile of deficits to that seen in FTD, with ALS patients exhibiting changes in social cognitive abilities. Gibbons et al. (2007) documented deficits on a Theory of Mind (ToM) task, where individuals with ALS displayed reduced ability to interpret cartoons and stories involving the comprehension of a character's mental state (e.g., false beliefs and deception). ALS patients exhibited profound impairments in deciphering the social component of this task in addition to comprehending humorous physical properties associated with the scene. It should be noted that poor performance on the ToM task correlated with deficits on a measure of executive functioning (i.e., the Wisconsin Card Sorting Task). Given that ToM tasks place strong demands on frontal networks, the authors hypothesized that executive dysfunction may contribute or be solely responsible for the poor social inferential and reasoning skills displayed within this ALS sample.

Meier et al. (2010) recently investigated social cognitive deficits in a small non-demented sample of ALS patients using the Faux Pas test. This ToM task required ALS patients to determine whether a character in a scenario had said something socially inappropriate. This task has been found to be sensitive in corroborating the presence of FTD and lesions in prefrontal networks (Stone et al., 1998; Gregory et al., 2002). A large proportion of this sample (9 of 18 patients) was unable to identify faux pas elements when compared to controls. However, there were no group differences on stories that did not contain faux pas components. Contrary to Gibbons et al. (2007), after co-varying out measures of verbal fluency, Meier et al.'s study found that executive dysfunction did not significantly impact performance on social cognitive tasks. Similar findings were reported by Girardi et al. (2011), who investigated ToM in ALS using a judgment preference task. This task required participants to infer the preference of another by means of eye-gaze direction as a social cue. Results of this study indicated that 64% of ALS patients were impaired on the attentionally demanding condition (i.e., when the distractor was present), while 36% performed poorly when the distractor was absent. Error analysis revealed that a subset of patients displayed a lack of inhibition and an egocentric tendency to select their own favored object while disregarding the one preferred by the face in the task.

Research (Abrahams, 2011) suggests that intact emotion processing is imperative to social cognition and perspective taking. Deficits in emotion processing are well-recognized in FTD and have been reported in ALS (Girardi et al., 2011). Studies have noted that tests of emotion recognition are sensitive to FTD-like pathology in ALS (Lillo et al., 2010; Woolley et al., 2011), however, research has produced mixed results. Deficits in emotion prosodic speech recognition have been reported (Zimmerman et al., 2007), however, these measures tend not to possess the discriminatory properties of facial emotion recognition tasks. In a recent study, ALS patients displayed deficits on two distinct measures involving the comprehension of simple (Facial Expressions of Emotions test) and complex (Reading the Mind in the Eyes test) displays of facial emotional expressions (Girardi et al., 2011). While ALS patients performed worse than controls on tests of emotion recognition, these differences were only marginally statistically significant, with the authors reporting that a subset of five patients (four bulbar, one limb onset) performed within abnormal ranges.

The static nature of social cognitive (e.g., Faux Pas test) and emotion recognition measures (e.g., Facial Expressions of Emotions Test) employed in ALS research to date fails to adequately represent the non-artificial and dynamic scenarios that are conveyed in real-world interactions, which encompass a combination of multifaceted expressions of emotional, prosodic, and facial morphology. The comprehension of sarcasm for instance, is a complex process that requires the appreciation of both the latent and actual content in order to evaluate both the facts surrounding the scenario and the inferences made by the speaker (McDonald, 1999). Individuals with ventromedial and orbitofrontal lesions, similar to regions implicated in social cognitive dysfunction in ALS (Meier et al., 2010), have been reported to perform poorly on tests of sarcasm recognition which has also been found to correlate with a reduction in empathy and inferring affect (Shamay-Tsoory et al., 2001). One may therefore anticipate similar deficits in patients with ALS.

Research using “real-world” measure of social cognition [The Awareness of Social Inference Test, (TASIT); see McDonald et al., 2003], conveyed through video vignettes of actors making sincere, sarcastic (e.g., a congruent statement relating to a specific event) or paradoxically sarcastic statements (e.g., a seemingly true, yet contradictory statement) has further characterized the scope of social cognitive difficulties within specific clinical populations. Studies have shown that patients with FTD and corresponding frontotemporal atrophy on MRI (Kipps et al., 2009), and semantic dementia (Rankin et al., 2009), display reduced ability, relative to controls, in interpreting “real-world” sarcastic statements on the TASIT. Analysis of imaging data from both studies suggests that atrophy in the right lateral orbitomedial—temporal lobe—amygdala network, but not orbitofrontal or dorsolateral neocortical networks, are related to deficits in deciphering sarcasm (Kipps et al., 2009; Rankin et al., 2009). As it stands, research in ALS has yet to explore and characterize the nature of social cognitive impairment by means of exploring the interpretation of real-world social interaction (i.e., body language, paralinguistic cues, and transient expressions of facial expression) and examine the potential impact that these impairments may conceivably place on ALS patients and caregivers.

We investigated the ability of patients with ALS to use paralinguistic cues to aid in the interpretation sarcasm by employing a real-world measure of social cognition. Unlike previous research examining social cognitive deficits in ALS, the stimuli in the present study are arguably more ecological than written sarcasm stimuli, and may effectively incorporate affective paralinguistic cues as to the speaker's real intention. Compared to previous studies that have employed static measures of social cognition, the TASIT incorporates dynamic social exchanges that can be considered more akin to real-life social situations. By employing such a tool, identified deficits may extrapolate more readily to real-world social functioning, thus facilitating early diagnosis and intervention with patients and their families.

The aim of this study was to add to the corpus of existing knowledge and further characterize the extent of social cognitive impairment in ALS by incorporating a more real-world measure of social cognition. It was hypothesized that non-demented ALS patients would perform similarly to controls on tests of basic emotion discrimination. It was also hypothesized that non-demented ALS patients would perform more poorly than controls in interpreting simple sarcastic and paradoxical sarcastic statements, but not sincere statements. Lastly, it was hypothesized that after controlling for difficulties on a task of cognitive flexibility (i.e., The Brixton Spatial Anticipation Task), individuals with ALS would still display significant difficulties in interpreting sarcastic stimuli.

## Method

### Participants

Participants were recruited from an Australian specialist multidisciplinary ALS clinic over a 10-month period, commencing in 2011 and concluding in 2012. For inclusion in the study, participants were required to have a diagnosis of clinically probable or definite ALS, as defined by the El Escorial criteria (Brooks et al., 2000). Participants with a past or present diagnoses of other neurological and/or psychiatric disease, including dementia or suspected FTD, were excluded, as were those with a history of head injury, or other medical illness known to affect central nervous system function. Non-English speakers or those with significant hearing impairment were also excluded from the study. Patients with ALS who had significant respiratory involvement (FVC < 70%) were also excluded. A total of 53 participants were initially recruited. Of these 53, 8 participants declined/withdrew form the study citing personal reasons. Of the remaining sample, 10 participants performed poorly on the Addenbrooke's Cognitive Examination Revised (i.e., two standard deviations below the control group mean) and were removed. A total of 35 non-demented participants with ALS were included in the study. Thirty age-and education-matched healthy controls were recruited via local community groups and sporting clubs. The exclusion criteria for healthy control participants were based on the criteria outlined above for ALS patients.

Informed consent was obtained from all participants. Ethical approval was obtained from the Calvary Health Care Bethlehem and the La Trobe University Human Research Ethics Committees.

## Materials

### Addenbrooke's cognitive examination revised (ACE-R)

To facilitate recruitment of a non-demented ALS sample, all participants completed the *Addenbrooke's Cognitive Examination Revised* (ACE-R; Mioshi et al., 2006), a brief cognitive screening tool with good reliability. The ACE-R examines attention/orientation, memory, verbal fluency, language and visuo-spatial abilities. Not all ALS participants were able to complete all aspects of the ACE-R. As a result, the language and visuo-spatial components of the ACE-R were modified to control for individual variations in bulbar and upper limb control. On the verbal fluency subtest, patients with ALS who were dysarthric and needed to provide a written response, were given an extra 60 s to complete the subtest to ensure that they were not penalized. This was a variation of the method employed by Abrahams et al. (2000). The maximum possible score for the modified ACE-R was 86. Given the study sought to include participants without dementia, the ACE-R was utilized solely to screen for poor cognitive performance. Participants were excluded from the study if they performed below the conservative statistical cut off of two standard deviations below the control group mean.

### The brixton spatial anticipation test

The *Brixton Spatial Anticipation Test* (Burgess and Shallice, 1997), a rule attainment task consisting of 56 pages of an identical array of ten circles, one of which is colored blue and changes location based on a logical pattern. Subjects are shown one page at a time, and are required to determine where the blue circle will appear on the consecutive pages by attempting to see a pattern or rule based on the information provided on the previous page. The Brixton is a validated measure with good reliability, and does not demonstrate hemispheric lateralization (Van den Berg et al., 2009).

### The awareness of social inference test (TASIT)

The Emotion Evaluation and Test of Social Inference (Minimal) subtests from *The Awareness of Social Inference Test (TASIT)* (McDonald et al., 2007) were used to assess comprehension of basic emotion and the ability to detect speaker intention, attitude, and meaning. The Emotion Evaluation subtest uses 14 professionally enacted video vignettes, with portrayals of positive (happiness, surprise, and neutral) and negative emotions (anger, disgust, fear, and sadness) lasting 10–20 s. Subjects were required to state the emotion portrayed by one of the actors in the vignette from a response card, which included the emotions in random order. Patients were queried as to their understanding of each emotion prior to commencing the task. Nine video vignettes of actors making sincere, sarcastic or paradoxically sarcastic statements were then shown to participants who were aware that they would subsequently be asked to endorse or reject a series of statements about what a specific actor was doing, saying, thinking, and feeling. All vignettes were shown on a 15-inch computer screen, with an attached loudspeaker system.

Examples of statements enacted by characters in video vignettes of the Tasist, either in a Sincere, Sarcastic, or Paradoxical Sarcastic tone is listed below.

*Sincere Statement (conveyed in a congruent manner)*

Ruth: “My sister is feeling really down. Would you mind if she came to the mountains with is this weekend?”

Gary: “I don't know why you have to ask. You know how I feel about your sister.”

Ruth: “She really needs to get away.”

Gary: “Don't we all. Why don't we invite some other people as also. What's your mother doing this weekend?”

Ruth: “No. I just want my sister to come.”

Gary: “Come on, we can make a family weekend of it”

*Sarcastic Statement (conveyed in a congruent statement relating to a specific event)*

Michael: “Well, congratulate me!”

Gary: “What for?”

Michael: “I've got a date with Anne.”

Gary: “Anne!”

Michael: “Come on, don't be jealous.”

Gary: “Sure, I'm jealous.”

*Paradoxical Sarcastic Statement (convey as a seemingly true, yet contradictory statement)*

Michael: “That was a massively long report you wrote. I could hardly lift it.”

Ruth: “Yes, just a few brief notes.”

Michael: “You poor thing. It must have taken you all weekend.”

Ruth: “Oh yes, I had a wonderful time. Just lying around, resting up after a hard week.”

### Symptoms of mood

Symptoms of mood disturbance were assessed using a modified version of the *Hospital Anxiety and Depression Scale* (HADS) for ALS patients (Gibbons et al., 2011). In this modified version, items pertaining to psychomotor activity were removed. The modified HADS is a validated 12-item self-report questionnaire with good reliability (Gibbons et al). Scores exceeding 9 indicate the presence of anxiety and scores exceeding 8 indicate the presence of depression.

### Functional impairment

Functional impairment was assessed using the *ALS Functional Rating Scale*-*Revised* (ALSFRS-R; Brooks et al., 2000), a validated rating instrument for monitoring the progression of disability in ALS which provides a physician-generated estimate of the patient's degree of functional impairment. Each item is rated on a five-point scale, ranging from 0 to 4. Individual item scores are summed to produce a reported score ranging from 0 to 40, with higher scores indicating better functioning.

### Procedure

Demographic information including gender, age, education, and past medical history was collected for all participants. For persons with ALS, information regarding ALS onset and diagnosis was also collected. ALS clinical phenotypes were determined by the treating neurologist.

Patients with ALS completed the ACE-R, Brixton, TASIT and the HADS. The ALSFRS-R was completed by the treating neurologist. All participation/data collection of ALS patients took place during home visits.

Control participants completed the ACE-R, Brixton and TASIT.

## Data analysis

### Demographic data

Demographic data were analyzed using analysis of variance (ANOVA; basic cognition, age, education, gender, duration of illness, anxiety, and depression).

### Tests of neuropsychological functioning

#### The brixton spatial anticipation task

Between groups comparisons on a task of cognitive flexibility, as measured by scores on the Brixton, were analyzed using ANOVA.

#### Scores on the emotion evaluation test

Due to sample size restrictions, between groups comparisons amongst individual emotions were unable to be explored.

Therefore, Tasit emotion recognition scores were combined to form two superordinate variables: positive (happy, surprised, and neutral) and negative (anger, disgust, fear, and sadness) which were then normalized to a maximum score of 1 per item. Group means on emotion recognition were compared by ANOVA.

#### Scores on the test of social inference (minimal)

Group differences on interpretation of sincere, simple sarcasm and paradoxical sarcasm were analyzed using ANOVA. The distributions of scores on simple and paradoxical sarcasm were negatively skewed and had to be reflected and natural-log transformed to meet the assumption of normality for parametric tests. Exploratory analysis between variables was carried out using analysis of covariance (ANCOVA).

## Results

### Demographic characteristics for ALS and controls participants

There was no significant difference between ALS patients and controls on a test of basic cognitive functioning as measured by the modified ACE-R [*F*_(1, 64)_ = 3.26, *P* ≥ 0.05]. There was no significant difference in levels of education χ^2^ [1, (*N* = 65) = 0.165, *P* ≥ 0.05], gender χ^2^ [1, (*N* = 65) = 0.154, *P* ≥ 0.05], or age [*F*_(1, 64)_ = 0.147, *P* ≥ 0.05] between ALS and control participants. Overall, ALS patients did not report high levels of depression or anxiety. The average time of disease duration from time of onset was 32 months—(please refer to Table 1).

**Table 1:** **Demographic data for group**.

**Variable**	**ALS (*N* = 35) mean (*SD*) range**	**Controls (*N* = 30) mean (*SD*) range**	***P*-Value**
Age (years)	63.51 (9.69)	62.63 (8.66)	>0.05
Gender			>0.05
Male *n* (%)	17 (48.6%)	10 (33.3%)	
Female *n* (%)	18 (51.4%)	20 (66.7%)	
Education			>0.05
Incompleted high	7 (20%)	10 (33.3%)	
school/trade *n* (%)			
Completed high	9 (25.7)	5 (16.7%)	
school *n* (%)			
Completed tertiary	19 (54.3)	15 (50%)	
education *n* (%)			
Months since symptom onset	32.05 (33.23)		
ALSFRS	36.4 (8.97)		
	12–47		
HADS Depression	3.52 (2.60)		
HADS Anxiety	5.97 (3.42)		

Note: ACE-R, Addenbrooke's Cognitive Examination Revised; ALSFRS-R, ALS Functional Rating Scale—Revised; HADS, Hospital Anxiety and Depression Scale.

### Tests of neuropsychological functioning

#### The brixton spatial anticipation task

There was a significant difference between groups on the Brixton, with ALS patients performing significantly worse on a task assessing cognitive flexibility compared to controls [*F*_(1, 64)_ = 16.74, *P* < 0.05, η^2^ = 0.21].

#### Emotion discrimination—negative vs. positive emotions

**Figure 1:**
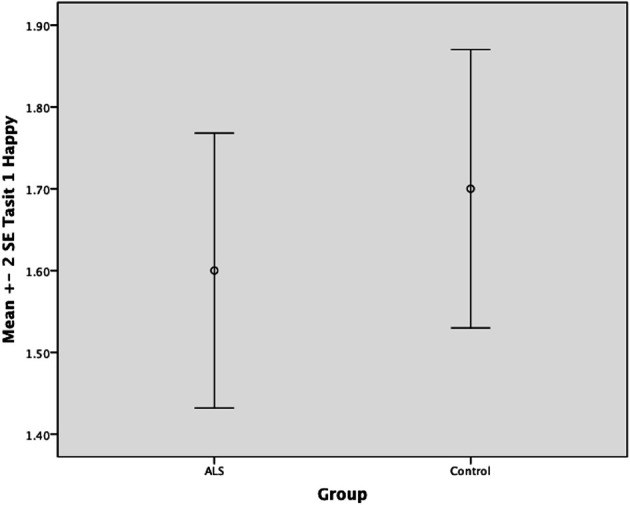
**Standard error of the mean for ALS and Control participants on Tasit Emotion Discrimination Happy**.

**Figure 2:**
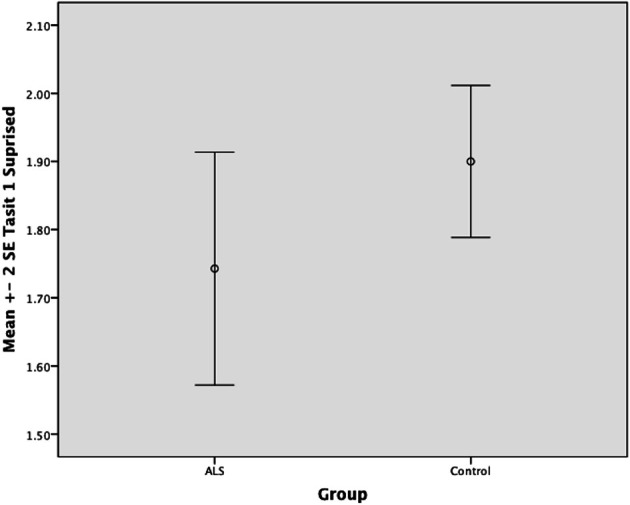
**Standard error of the mean for ALS and Control participants on Tasit Emotion Discrimination Surprised**.

**Figure 3:**
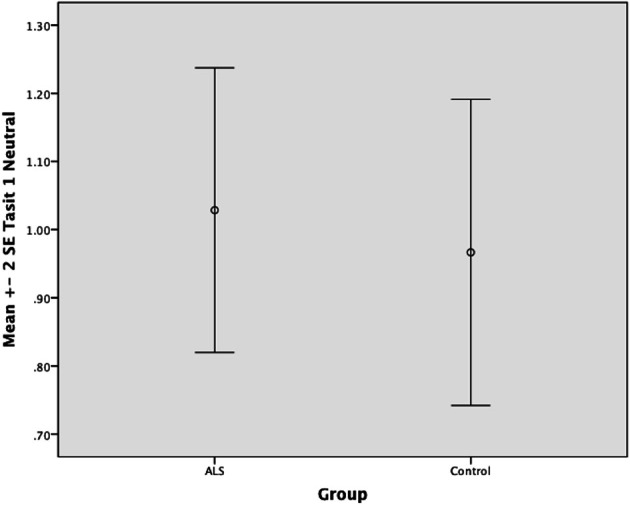
**Standard error of the mean for ALS and Control participants on Tasit Emotion Discrimination Neutral**.

**Figure 4:**
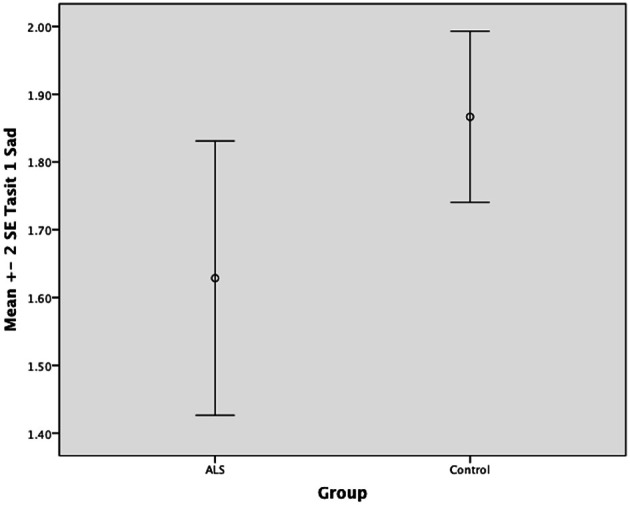
**Standard error of the mean for ALS and Control participants on Tasit Emotion Discrimination Sad**.

**Figure 5:**
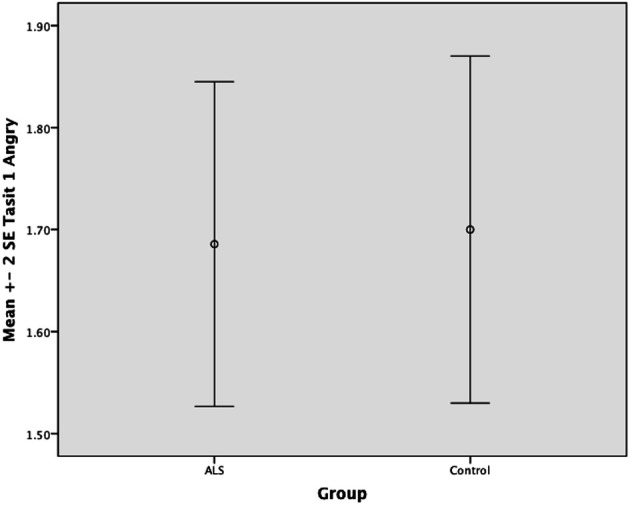
**Standard error of the mean for ALS and Control participants on Tasit Emotion Discrimination Angry**.

**Figure 6:**
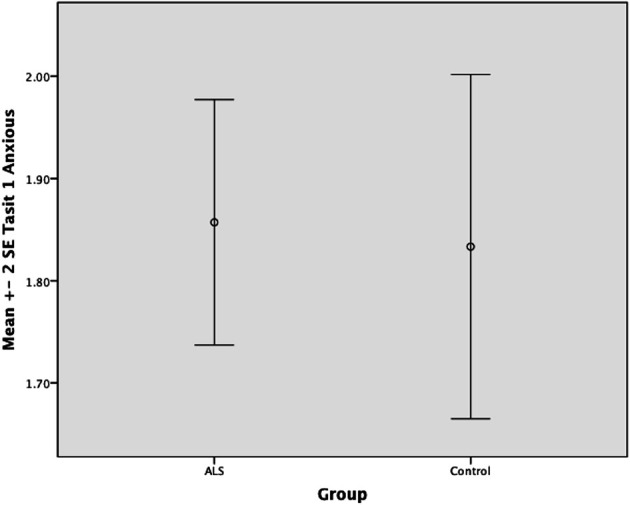
**Standard error of the mean for ALS and Control participants on Tasit Emotion Discrimination Anxious**.

**Figure 7:**
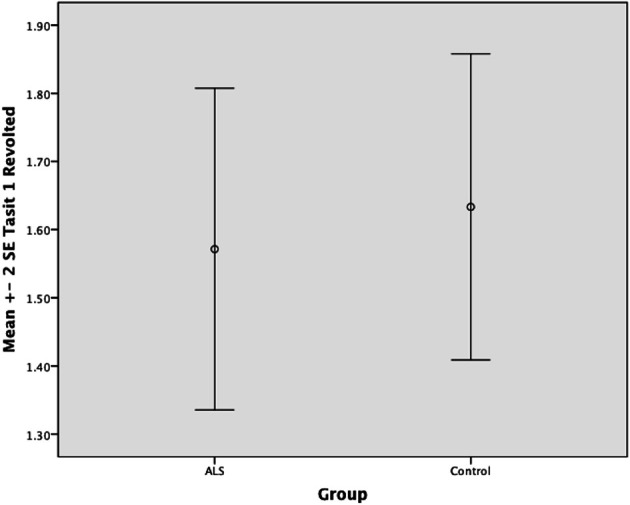
**Standard error of the mean for ALS and Control participants on Tasit Emotion Discrimination Revolted**.

Overall, there was no significant difference between ALS patients and controls in their ability to recognize positive emotions (happy, surprised, and neutral) [*F*_(1, 64)_ = 0.759, *P* ≥ 0.05], or negative emotions (anger, disgust, fear, and sadness) [*F*_(1, 64)_ = 0.663, *P* ≥ 0.05] (please refer to Figures 1–7).

#### Test of social inference

**Figure 8:**
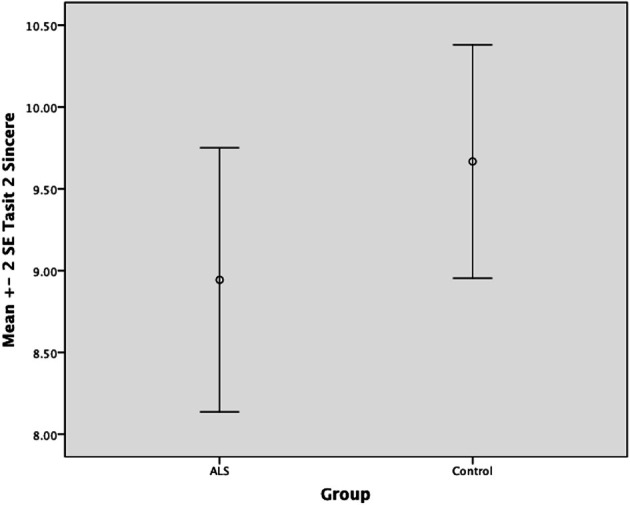
**Standard error of the mean for ALS and Control participants on Tasit Social Inference Sincere Statement**.

**Figure 9:**
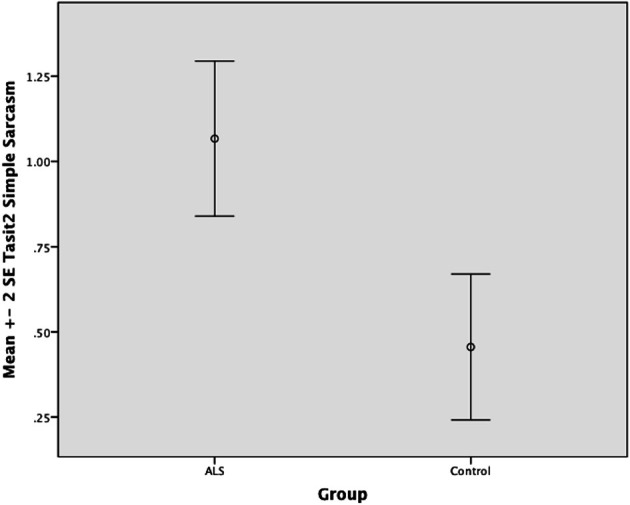
**Standard error of the mean for ALS and Control participants on Tasit Social Inference Sarcastic Statement**.

**Figure 10:**
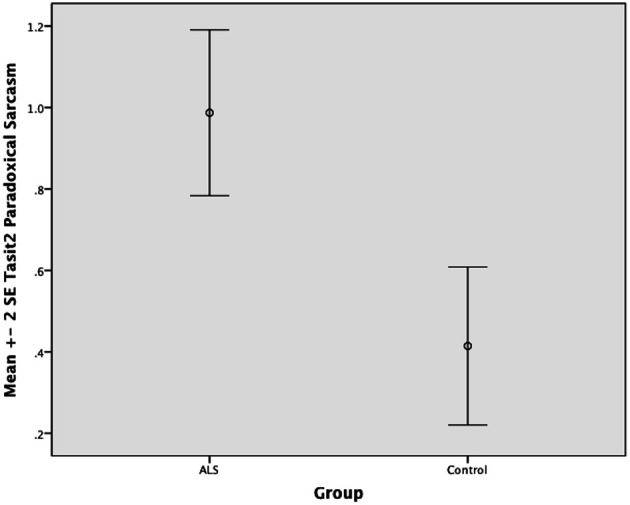
**Standard error of the mean for ALS and Control participants on Tasit Social Inference Paradoxical Sarcastic Statement**.

There was no significant difference between ALS patients and controls in the ability to recognize sincere statements [*F*_(1, 64)_ = 1.75, *P* ≥ 0.05]. However, ALS patients displayed a significant impairment in interpreting simple sarcastic statements [*F*_(1, 64)_ = 15.00, *P* ≤ 0.05, η^2^ = 0.19] and paradoxical sarcastic statements [*F*_(1, 64)_ = 16.26, *P* ≤ 0.05, η^2^ = 0.20] compared to healthy controls. Further analysis revealed no significant difference between ALS phenotypes (bulbar vs. limb onset) on measures of interpreting simple sarcastic statements [*F*_(1, 34)_ = 3.95, *P* ≥ 0.05], or paradoxical sarcastic statements [*F*_(1, 34)_ = 0.32, *P* ≥ 0.05] (please refer to Figures 8–10).

To determine whether difficulties with interpreting simple sarcastic and paradoxical sarcastic statements within the ALS group were related to levels of executive difficulty, ANCOVA was conducted. After controlling for executive difficulty as measured by scores on the Brixton, results suggest that the ALS group still performed poorly in interpreting simple sarcastic statements [*F*_(1, 64)_ = 10.35, *P* ≤ 0.05] and paradoxical sarcastic statements [*F*_(1, 64)_ = 8.74, *P* ≤ 0.05]—(please refer to Table 2).

**Table 2:** **Neuropsychological data for ALS and control participants**.

**Variable**	**ALS (*N* = 35) mean (*SD*)**	**Controls (*N* = 30) mean (*SD*)**	**η2**	***P*-Value**
ACE-R	79.60 (3.64)	81.23 (3.61)		>0.05
Brixton	20.02 (8.06)	13.43 (3.86)^*^	0.21	<0.05
**EMOTION DISCRIMINATION**				
Positive emotions	4.40 (1.06)	4.6 (0.723)	0.012	>0.05
Negative emotions	6.77 (1.28)	7.00 (0.909)	0.01	>0.05
**TEST OF SOCIAL INFERENCE**				
Sincere	8.94 (2.38)	9.66 (1.95)	0.027	>0.05
Simple sarcasm^rnl^	9.40 (2.46)	11.10 (1.32)^*^	0.192	<0.05
Paradoxical sarcasm^rnl^	9.82 (1.85)	11.23 (1.16)^*^	0.205	<0.05

Note:

* p <0.05; rnl, median and interquartile range; Brixton, Brixton Spatial Anticipation Test.

## Discussion

To our knowledge, this is the first study to investigate social cognitive and emotion recognition deficits in non-demented ALS patients using a measure of social cognition conveyed via video vignettes. Consistent with previous research (Meier et al., 2010), this non-demented cohort of ALS patients recruited from a specialist outpatient Motor Neuron Disease clinic showed evidence of reduced cognitive flexibility. Relative to the age and education matched control group, the ALS group performed significantly worse on a task of cognitive flexibility (i.e., the Brixton Spatial Anticipation Task) and tasks requiring individuals to make higher order social inferences in the realm of sarcasm. However, as expected the ALS group performed similar to controls on lower order tasks of basic emotion discrimination and making inferences regarding sincere statements.

The ability to accurately discriminate and respond appropriately to a range of emotion types is an important aspect of social behavior that reinforces, guides and influences future actions. In this study, the ALS group performed similarly to controls, displaying an intact ability to accurately discriminate between a range of positive and negative emotions. Previous research into affect recognition in ALS has produced mixed results, with small percentages of patients displaying significant difficulties in identifying facial expressions of emotion (Girardi et al., 2011) or deficits in emotional prosodic speech recognition (Zimmerman et al., 2007; Meier et al., 2010). Given that past studies have employed a wide range of stimulus modalities and tasks, this inconsistent finding is not surprising. However, in this instance it can be said that this sample of ALS patients did not display deficits in recognizing either positive or negative emotions relative to controls.

In the current study, the ALS group did not display any difficulty, relative to controls, in comprehending sincere statements. While not comprehensively explored in previous ALS research, this pattern of results has been found in other clinical groups with frontal dysfunction, such as bvFTD (Kipps et al., 2009) and Semantic Dementia (Rankin et al., 2009). Moreover, these findings on the TASIT appear generally consistent with Meier et al.'s (2010) results, where ALS patients displayed an inability to recognize faux pas elements within a story yet performed similarly to controls when intentions were overtly obvious and did not require interpretation of faux pas elements. Overall, it appears that the present sample of ALS patients display intact ability to discern simple exchanges between individuals so long as they are not overly complex.

Unlike the ability to interpret rudimentary social exchanges, such as sincere statements, the ability to accurately and effectively interpret higher levels of social cues, such as sarcastic exchanges, requires an understanding of paralinguistic cues (McDonald et al., 2003). The ability to appreciate and discern complex social exchanges requires an individual to evaluate a situation using a combination of multifaceted expressions of emotional, prosodic and facial morphology, while at the same time having the social nous to decipher the covert from overt within the social exchange. In the current study, impaired performance on both sarcastic and paradoxical sarcastic statements in this non-demented ALS group was prominent. Findings such as these have been conveyed in past research (Meier et al., 2010), where non-demented ALS patients display intact cognitive function on general screening tools, however, display a reduced ability to make judgments and social inferences on a host of tasks thought to be sensitive to social cognitive impairment (Meier et al.). Kipps et al. (2009) reported that individuals with FTD, and corresponding frontotemporal atrophy on MRI, were significantly worse at deciphering sarcastic vs. sincere statements on the TASIT. This profile of impairment strongly suggests that a proportion of ALS patients do indeed display social cognitive deficits similar to that reported in FTD.

Controversy remains regarding the precise role that executive functions play in effectively deciphering socially challenging situations. In the current study, the ALS group performed worse than controls on a test of executive functioning—once again confirming the presence of this widely reported cognitive hallmark (Gibbons et al., 2008). However, after co-varying the effects of executive difficulties out, the ALS group still performed poorly when recognizing both sarcastic and paradoxical sarcastic statements (that is, tasks tapping higher order social cognitive abilities). This finding is consistent with Meier et al.'s (2010) findings, where after co-varying out performance on an executive function task (verbal fluency), participants with ALS were still impaired at recognizing faux pas elements and making inferences based on the scenario. Regardless, executive dysfunction in ALS strongly suggests frontotemporal cortical involvement (Gibbons et al., 2008; Hodges, 2008).

The recent findings of Elamin et al. (2011) suggest that in patients without dementia, the presence of executive dysfunction is a negative prognostic indicator. The authors argue that compromised executive functions may be a marker for a more aggressive, and distinct form of ALS with a significantly shorter period of survivorship. It seems likely that distinctive social cognitive processes involve executive functioning to varying magnitudes. It may be that the impact of executive functioning on social cognitive processes can be conceptualized as facilitating, rather than delineating, one's ability to accurately decipher social cognitive stimuli. In this instance, it can be argued that the difficulty exhibited by persons with ALS in deciphering socially challenging stimuli may in fact be related to compromised executive faculties. The Brixton Spatial Anticipation Task purports to measures a person's ability to detect a rule, to follow it, and to switch to a new rule. Such aspects of cognitive flexibility are essential when interacting in social situations. In cases where individuals fail to utilize and impose such aspects of cognitive flexibility in social scenarios, they are likely to encounter significant difficulty when interpreting dynamic social exchanges, fail to process information in a timely fashion and respond accordingly, exhibit difficulty linking new information to prior knowledge, and experience frustration due to a lack of understanding of what has transpired.

The current study did have a number of limitations. Although this sample of ALS participants did not display impairments in basic emotion recognition as measured by scores on the TASIT, it is possible that the artificial nature of the videos vignettes may have overly caricaturized emotional states in comparison to natural expressions of emotions. Thus, a null finding may have occurred when impairments were indeed present. However, this null finding is consistent with the majority of the literature examining basic emotion discrimination in ALS to date. It is also acknowledged that the lack of fine gradations of transient expression of facial emotion and lack of incorporation of reaction times may provide additional cues that facilitate basic emotion discrimination. This does appear to be a consistent limitation in the current ALS emotion recognition research and warrants immediate attention.

This current study only employed a single measure of executive functioning (i.e., Brixton Spatial Anticipation Task), and as a result may have underestimated the relationship that executive faculties play in deciphering socially challenging content. By employing a wider range of executive tasks in this current study, we may have uncovered a direct relationship amid social cognitive deficits and executive functions. However, selecting an appropriate host of executive measures that circumvent both speech and physical limitations when conducting research with ALS population continues to be challenging.

Furthermore, we acknowledge that using the modified ACE-R to aid in screening for cognitive impairment was a limitation in this study. However, given the difficulty of conducting cognitive assessments within a population that suffers from speech difficulties and/or upper limb dysfunction, the use of this measure to facilitate the exclusion of participants with reduced cognitive functioning was necessary.

In summary, the key findings of this study indicate that non-demented ALS patients displayed deficits in deciphering sarcastic and paradoxical sarcastic statements which are thought to be related to cortical degradation in the orbitofrontal and/or dorsomedial prefrontal regions (Meier et al., 2010). While research (Abrahams et al., 2005; Gibbons et al., 2008) has reported that a proportion of ALS patients develop frontal lobe dementia which ultimately results in profound behavioral dysinhibition and executive dysfunction, the recognition that subtle alterations in these cognitive functions present in a proportion of non-demented ALS patients has now been recognized (Meier et al., 2010). While not yet specifically documented in ALS, autism research has ascribed high-level social cognitive deficits as leading to reduced responsiveness to others and a lack of social and emotional reciprocity (Chevallier et al., 2012). This lack of social perceptiveness and reciprocity is especially poignant in ALS, where social relationships become paramount in the context of reduced speech and severe physical disability.

The inability to read social cues and effectively communicate has the potential to place significant amounts of strain on familial/interpersonal relationships. This is especially true for ALS carers who are placed under large amounts of stress due to the physically debilitating nature of the disease (Gordon et al., 2007). It is highly likely that as alterations in social cognition become more prominent, ALS patients will experience difficulties in being able to engage in a wide range of activities that, in turn, reduces their quality of life. Therefore, the detection of subtle alterations in cognition amongst ALS patients in early stages of the disease is paramount to inform future decision-making and advanced care planning.

### Conflict of interest statement

The authors declare that the research was conducted in the absence of any commercial or financial relationships that could be construed as a potential conflict of interest.
